# Dietary Bazhen San solid-state fermentation product improves laying performance, immunity and intestinal health in laying hens during the late laying period

**DOI:** 10.3389/fimmu.2025.1673604

**Published:** 2026-03-24

**Authors:** Zenghui Zhao, Xiao Wang, Yongzhan Bao, Jiahui Meng, Jinlong Gong, Lu Zhang, Zhaonian Li, Weiyu Yao, Yanan Chuo, Wanyu Shi, Jianxi Li

**Affiliations:** 1 College of Traditional Chinese Veterinary Medicine, Hebei Agricultural University, Baoding, China; 2 Hebei Key Laboratory of Traditional Chinese Veterinary Medicine, Baoding, China; 3 Lanzhou Institute of Husbandry and Pharmaceutical of Chinese Academy of Agricultural Sciences, Lanzhou, China

**Keywords:** Bazhen San solid-state fermentation product, laying hens, immunity, intestine, microbiota

## Abstract

The aim of this study was to investigate the effects of solid-state fermentation products of Bazhen San (FB) on the production performance, immunity, and intestinal health of laying hens during the late laying stage. A total of 150 70-week-old laying hens were randomly assigned to five treatment groups, with five replicates per group and six hens per replicate. The control group (CON) was fed a corn–soybean meal-based diet, whereas the other four treatment groups were supplemented with 0.3% FB (LFB), 0.6% FB (MFB), 0.9% FB (HFB), and 0.6% unfermented Bazhen San (BZ), respectively. The results showed that, compared with the CON group, all treatments significantly increased the egg production rate and reduced the feed-to-egg ratio (*P* < 0.05). Moreover, the effect in the MFB group was significantly greater than that in the BZ group (*P* < 0.05). In terms of egg quality, the MFB and HFB groups significantly improved yolk color and Haugh units (*P* < 0.05). Regarding immune function, serum immunoglobulin A (IgA) levels were significantly increased in all treatment groups (*P* < 0.05), whereas interleukin-1β (IL-1β) and interleukin-6 (IL-6) concentrations were significantly decreased (*P* < 0.05). The MFB and HFB groups also significantly increased serum immunoglobulin G (IgG) levels (*P* < 0.05), as well as jejunal and ileal secretory immunoglobulin A (sIgA) levels (*P* < 0.05). In addition, serum IgG levels in the MFB group were significantly higher than those in the BZ group (*P* < 0.05). In terms of intestinal health, FB treatment significantly enhanced antioxidant enzyme activity in the jejunum and ileum, reduced malondialdehyde (MDA) content, improved intestinal morphology. The microbiome analysis of the cecum showed that FB improved the abundance of beneficial bacteria in the intestine. Spearman correlation analysis revealed that the relative abundance of *Odoribacter* and *Enterococcus* was positively correlated with serum IgA levels and negatively correlated with IL-6 concentration. Therefore, dietary supplementation with FB can improve intestinal health, and systematically improve the immune status of the body, thereby promoting the health of laying hens during the late laying stage and improving production performance, dietary 0.6% to 0.9% FB inclusion is suggested.

## 1 Introduction

After the peak laying period has passed, the extending laying cycle leads to significant nutritional depletion and physiological stress in laying hens. This subsequently results in declined bodily functions, immune imbalance, intestinal damage, reduced laying performance, and poor egg quality during the late laying phase, all of which seriously reduce economic benefits (1). The immune system serves as the primary defense against diseases, when it becomes compromised, the risks of pathogen invasion and disease increase (2). Furthermore, sustained egg production and chronic pathogen exposure place high demands on the digestive system, particularly the intestinal tract. This results in impaired intestinal mucosal barrier function, gut microbiota dysbiosis, reduced feed digestion efficiency, and heightened risks of harmful substances entering the systemic circulation (3). Collectively, these changes undermine hen health and shorten productive lifespans. In the context of antibiotic bans, natural bioactive additives such as traditional Chinese medicine (TCM) and probiotics have gained attention for their benefits in improving immunity and intestinal health in laying hens (4–6).

Bazhen San is a classical TCM formula comprising *Codonopsis Radix*, *Atractylodis Macrocephalae Rhizoma*, *Poria*, *Glycyrrhizae Radix*, *Angelicae Sinensis Radix*, *Chuanxiong Rhizoma*, *Rehmanniae Radix Praeparata*, and *Paeoniae Radix Alba*. It acts to tonify qi and blood while also improving organ function (7). Research has shown that BZD enhances immunity and inhibits tumor progression in mouse models of colorectal cancer (7), in addition to exerting anti-inflammatory and antioxidant effects (8). Modified Bazhen San (substituting Codonopsis with Astragalus and Jujube) has been shown to improve sow immunity and gut microbiota (9). Furthermore, dietary supplementation with 0.5%-1% Bazhen San has been reported to enhance serum IgG and γ-globulin levels and improve antioxidant capacity in broilers (10). Current extraction methods for the active ingredients in Bazhen San primarily rely on traditional techniques, including water extraction and alcohol extraction. However, these methods often have drawbacks such as low extraction efficiency, high energy consumption, and poor stability of the active components.

TCM fermentation technology utilizes microbial processing under controlled conditions to enhance the efficacy and bioavailability of herbal compounds (11). For instance, after fermentation, the content of polysaccharides, flavonoids, and saponins in astragalus significantly increases (12). Fermented TCMs have been shown to boost immunity, modulate gut microbiota, and improve health (13, 14). For instance, *Lactobacillus plantarum* fermented Shenling Baizhu San significantly enhances broiler immunity, intestinal barrier function, and microbiota diversity (15). While solid-state-fermented Bazhen San could theoretically benefit late-phase laying hens, limited research exists on its effects. This study therefore evaluates the impacts of varying doses of solid-state-fermented Bazhen San on immune function, intestinal morphology, barrier integrity, and microbiota composition in late-phase laying hens. The aim is to determine the optimal feeding dose and to provide a theoretical and practical basis for its application as a novel functional feed additive in poultry production.

## 2 Materials and methods

### 2.1 Drugs and reagents

The Chinese herbal medicines *Codonopsis Radix*, *Atractylodis Macrocephalae Rhizoma*, Poria, *Glycyrrhizae Radix*, *Rehmanniae Radix Praeparata*, *Angelicae Sinensis Radix*, *Stir-fried Paeoniae Radix Alba* and *Chuanxiong Rhizoma* were purchased from Qifu Traditional Chinese Medicine Sales Co., Ltd. (Anguo City, Hebei Province, China). *Lactobacillus plantarum* (JYLP-326) and *Bifidobacterium animalis* (JYBR-190) were provided by Shandong Zhongke Jiayi Bioengineering Co., Ltd. (China).

### 2.2 Preparation of Bazhen San solid-state fermented product

According to the 2020 edition of the Chinese Pharmacopoeia, Bazhen San is composed of *Codonopsis Radix*, *Atractylodis Macrocephalae Rhizoma*, *Poria*, *Glycyrrhizae Radix*, *Rehmanniae Radix Praeparata*, *Angelicae Sinensis Radix*, *Stir-fried Paeoniae Radix Alba*, and *Chuanxiong Rhizoma* at a ratio of 1:1:1:0.5:1.5:1.5:1:0.75.

The above herbs were washed to remove impurities and dried at 40°C. They were then mixed in the specified ratio, crushed, and passed through a 40-mesh sieve. Subsequently, 10% wheat bran and 10% soybean meal were added and thoroughly homogenized with the herbal powder. A mixed starter culture (*Lactobacillus plantarum* to *Bifidobacterium animalis* subsp. lactis at a 1:1 ratio) was incorporated into the mixture. The blend was then packed into 23 cm × 30 cm one-way venting fermentation bags, vacuum-sealed, and fermented under controlled conditions (37°C, 50% moisture, 1×10^9^ CFU/g inoculum) for 48 h to obtain the solid state fermented product of Bazhen San (FB).

### 2.3 Analysis of medicinal bioactive ingredients in FB

A 1.0 g sample of the Bazhen San solid-state fermented product was weighed and transferred to a round-bottom flask. 40 mL of distilled water was added, and the extraction was prepared in triplicate. The mixture was subjected to ultrasonic-assisted extraction for 20 min, after which the polysaccharide extract was collected. A standard curve was established using glucose as the reference standard. The ultraviolet (UV) absorbance of the extracts at 490 nm was measured using a UV-Vis spectrophotometer (DNM-9606, Beijing Pulang New Technology Co., Ltd., Beijing, China) according to the phenol-sulfuric acid method (16). For total flavonoid content analysis, a 1.0 g sample of the fermented product was weighed into a round-bottom flask, mixed with 20 mL of 60% (v/v) ethanol, and prepared in triplicate. After ultrasonic-assisted extraction for 20 min, the supernatant was collected, then adjusted to a final volume of 20 mL. The content of total flavonoids in FB was determined using the NaNO2-Al (NO3)3 color developing method, with rutin as the reference standard. The UV absorbance at 510 nm was measured using the same spectrophotometer (17).

### 2.4 Experimental groups and management

The experimental protocol was approved by the Animal Protection and Use Committee of Hebei Agricultural University (Approval Number: 2022161). The trial was conducted in the animal laboratory of Hebei Agricultural University. A total of 150 healthy 70-week-old Jingfen laying hens with comparable egg production rates and body weights were randomly allocated into 5 treatment groups, with 5 replicates per group and 6 hens per replicate. The control group (CON) was fed a corn-soybean meal basal diet, while the other four groups received the basal diet supplemented with 0.3% Bazhen San solid-state fermented product (LFB group), 0.6% Bazhen San solid-state fermented product (MFB group), 0.9% Bazhen San solid-state fermented product (HFB group), or 0.6% non-fermented Bazhen San (BZ group). All hens had free access to feed and water throughout the experiment. A 2-week adaptation period was implemented, followed by an 8-week experimental period. Eggs were collected daily in the afternoon. All diets were formulated to meet nutrient requirements (**Table 1**).

**Table 1:** Composition of comparative feed (%, dry matter).

Ingredients	Content	Nutrient levels^2^	Content
Corn	63.30	ME (MJ/kg)	11.09
Soybean meal	25.37	Crude protein (%)	16.17
Limestone	8.50	Ca (%)	3.45
Sodium chloride	0.30	Available P (%)	0.36
Calcium hydrogen phosphate	1.90	Lys (%)	0.78
Premix^1^	0.32	Met (%)	0.36
L-lysine monohydrochloride	0.11		
DL-Methionine	0.20		
Total	100		

^1^Provided per kilogram of diets: vitamin A, 12,500 IU; vitamin D3, 2,500 IU; vitamin E, 30 IU; vitamin K3, 1.70 mg; vitamin B1, 2 mg; vitamin B2, 6 mg; vitamin B12, 0.025 mg; niacin, 36 mg; biotin, 0.2 mg; folic acid, 1.25 mg; calcium pantothenate, 13 mg; iron, 80 mg; copper, 8 mg; manganese, 100 mg; zinc, 75 mg; iodine, 1.0 mg and selenium, 0.15 mg.

^2^Nutrient levels were calculated values.

### 2.5 Sample collection

At the end of the 8-week experimental period and following a 12-hour fast, fifteen hens from each group were randomly selected, and blood samples were collected from the wing vein using serum separation tubes. The blood was centrifuged at 3,000 r/min for 10 minutes at 4°C, and the supernatant was transferred to sterile centrifuge tubes and stored at -20°C. After blood collection, the hens were anesthetized by intravenous injection of 50 mg/kg of pentobarbital sodium and euthanized by cervical dislocation after 2 minutes of anesthesia (18). Cecal contents were aseptically collected into sterile cryovials, flash-frozen in liquid nitrogen, and stored at -80°C. For intestinal morphology analysis, two segments of the jejunum and ileum were excised and immersed in 4% paraformaldehyde fixative. Approximately 2.0 g of intestinal mucosa was gently scraped from the intestinal wall using sterile microscope slides, wrapped in sterile aluminum foil, flash-frozen in liquid nitrogen, and stored at -80°C for subsequent analyses.

### 2.6 Determination of production performance and egg quality

The total number of eggs laid, egg weight, and the counts of unqualified eggs (including sand-shelled, soft-shelled, and broken) were recorded daily during the trial. Weekly summary calculation of feed consumption, and the feed-to-egg ratio for each replicate was calculated by dividing total feed consumption by total egg weight.

At the 8th week of the experiment, thirty eggs were randomly selected from each group for quality assessment, which included the following indices: egg shape index, eggshell strength, eggshell thickness, yolk color, and Haugh units. The long and short diameters of the eggs were measured using an egg form coefficient measuring instrument (NFN385, FHK Corp., Tokyo, Japan). The egg shape index was calculated by the ratio of long diameter to short diameter. The KQ-1A Eggshell Strength Tester (Beijing Tianxiang Feiyu Technology Co., Ltd., China) was employed to measure eggshell strength. An automatic egg quality analyzer (Robotmation Co., Ltd., Japan) was utilized to assess yolk color and Haugh units. To determine eggshell thickness, the shell membrane was carefully removed from the eggshell. Measurements were then taken at 3 specific points on the eggshell: the blunt end, the middle, and the sharp end. The average of these three measurements was calculated to establish the eggshell thickness value. This measurement was conducted using a micrometer.

### 2.7 Determination of serum immune indices

Serum concentrations of immunoglobulins (IgA, IgG, IgM) and cytokines interleukin-1 (IL-1), interleukin-6 (IL-6), interleukin-10 (IL-10), and tumor necrosis factor-alpha (TNF-α) were measured using commercially available ELISA kits (Yuanju Biotechnology Center, Shanghai, China) following the manufacturer’s instructions.

### 2.8 Determination of intestinal immune indices, permeability, and antioxidant parameters

The jejunal and ileal mucosa were homogenized in pre-cooled physiological saline solution (1:9, w/v) and centrifuged at 3,000 r/min for 10 min. The supernatant was collected for analysis. The concentrations of secretory immunoglobulin A (sIgA), zonula occludens-1 (ZO-1), and occludin were measured using ELISA kits (Yuanju Biotechnology Center, Shanghai, China) following the manufacturer’s protocols. Total antioxidant capacity (T-AOC), superoxide dismutase (SOD), glutathione peroxidase (GSH-Px), catalase (CAT), and malondialdehyde (MDA) levels were quantified using commercially available kits (Nanjing Jiancheng Bioengineering Institute, Nanjing, China). The protein concentration in the supernatant was determined using a BCA protein assay kit (Shanghai, China).

### 2.9 Determination of intestinal morphology

The morphological structure of jejunal and ileal tissues was evaluated using hematoxylin and eosin (H&E) staining. Fixed tissues preserved in 4% paraformaldehyde solution were trimmed, embedded, sectioned, deparaffinized, stained, dehydrated, and mounted for microscopic examination. Qualified specimens were selected for analysis. Villus height (VH) and crypt depth (CD) of the jejunum and ileum were measured using SlideViewer software, and the villus height-to-crypt depth ratio (VH/CD) was calculated. Five intact villi were measured per sample to ensure statistical reliability.

### 2.10 Analysis of cecal microbiota

Microbial DNA was extracted from the cecal contents of laying hens using the HiPure Stool DNA Kit (Model D3141, Magen Biotechnology Co., Ltd., Guangzhou, China) following the manufacturer’s instructions. The purity and integrity of nucleic acid samples were evaluated using a NanoDrop microspectrophotometer and agarose gel electrophoresis. The V3-V4 hypervariable regions of the bacterial 16S rRNA gene were amplified by PCR with the primer pair 341F (CCTACGGGNGGCWGCAG) and 806R (GGACTACHVGGGTATCTAAT). The PCR products were purified using AMPure XP Beads, quantified with a Qubit 3.0 Fluorometer, and used for library construction with the Illumina DNA Prep Kit. Library quality was verified using the ABI StepOnePlus Real-Time PCR System prior to paired-end sequencing (PE250) on an Illumina NovaSeq 6000 platform.

### 2.11 Statistical analysis

Data are presented as means ± standard error of the mean (SEM). One-way analysis of variance (ANOVA) followed by Tukey’s test was performed to compare differences among groups using IBM SPSS Statistics 26. Orthogonal polynomial contrasts were used to evaluate the linear and quadratic responses to increasing doses of FB across the CON, LFB, MFB, and HFB groups. A p-value < 0.05 was considered statistically significant. Figures were generated using GraphPad Prism 8.0.2. Spearman’s correlation analysis was conducted to evaluate associations between microbial composition and intestinal morphology/immunity indices.

## 3 Results

### 3.1 Medicinal bioactive ingredients of FB

After solid-state fermentation with *Lactobacillus plantarum* and *Bifidobacterium animalis* subsp. Lactis, the Bazhen San fermented product, contained crude polysaccharides at 36.82 ± 0.52 mg/g and total flavonoids at 13.02 ± 0.07 mg/g.

### 3.2 Effects of FB on egg production performance of late laying hens

As shown in **Table 2**, The dietary supplementation with FB and BZ treatment groups significantly exhibited higher egg-laying rates compared to the CON group (*P* < 0.05), accompanied by decreased feed-to-egg ratio (*P* < 0.05). Notably, the feed-to-egg ratio of the MFB group was significantly lower than that of the BZ group (*P* < 0.05). Nevertheless, there were no significant effects observed on the occurrences of unqualified egg rate and average egg weight (*P* > 0.05). The results demonstrated that supplementing the diet with FB and BZ improved the egg production rate in laying hens. Furthermore, compared with the BZ-supplemented group, the MFB group was more effective in reducing feed costs and enhancing feed conversion efficiency.

**Table 2:** Effects of FB on egg production performance of late laying hens.

Item	Group					SEM	P value		
	CON	LFB	MFB	HFB	BZ		A	L	Q
Egg laying rate, %	58.38^b^	70.78^a^	75.51^a^	77.27^a^	68.95^a^	1.487	< 0.05	< 0.05	< 0.05
Unqualified egg rate, %	9.5	7.416	7.236	6.619	7.479	0.398	0.193	< 0.05	0.071
Egg weight, g	56.8	60.38	62.93	60.63	60.88	0.688	0.068	< 0.05	< 0.05
Feed-to-egg ratio	2.82^a^	2.39^b^	2.21^c^	2.23^c^	2.40^b^	0.06	< 0.05	< 0.05	< 0.05

Data were expressed as means ± SEM (n = 30).

SEM, Pooled standard error of the mean.

^a–c^Means, within each row with different superscripts are statistically significantly different (*P* < 0.05).

A, P value of one-way ANOVA; L, P value of linear analysis; Q, P value of quadratic analysis.

### 3.3 Effects of BZ on the egg quality of late laying hens

As shown in **Table 3**, FB supplementation during late-phase egg production did not affect egg shape index, Shell strength, or Shell thickness throughout the experiment. Contrast to the CON group, the yolk color was significantly increased (*P* < 0.05) in the MFB and HFB groups. The yolk color of the MFB group was significantly higher than that of the BZ group (*P* < 0.05). Dietary supplementation with 0.9% FB significantly elevated Haugh unit compared to the CON group (*P* < 0.05). These results indicate that although FB supplementation did not alter eggshell quality parameters, it significantly improved yolk color and albumen quality. These findings indicate that FB can enhance the market value of eggs by improving key internal quality traits.

**Table 3:** Effects of FB on the egg quality of late laying hens.

Item	Group					SEM	P value		
	CON	LFB	MFB	HFB	BZ		A	L	Q
Egg shape index	1.31	1.34	1.33	1.32	1.33	0.006	0.593	0.524	0.782
Shell strength, N/m^2^	34.38	35.85	37.9	36.83	35.27	1.284	0.935	0.682	0.838
Shell thickness, mm	0.31	0.33	0.34	0.34	0.33	0.006	0.633	0.312	0.513
Yolk color	5.5^b^	7.5^ab^	7.67^a^	8.33^a^	7.00^b^	0.273	< 0.05	< 0.05	< 0.05
Haugh unit	63.92^b^	71.00^ab^	72.88^ab^	75.91^a^	69.62^ab^	1.166	< 0.05	< 0.05	< 0.05

Data were expressed as means ± SEM (n = 30).

SEM: Pooled standard error of the mean.

^a,b^Means within each row with different superscripts are statistically significantly different (*P* < 0.05).

A, P value of one-way ANOVA; L, P value of linear analysis; Q, P value of quadratic analysis.

### 3.4 The effect of FB on the immune function of laying hens in the late stage of egg production

**Figure 1:**
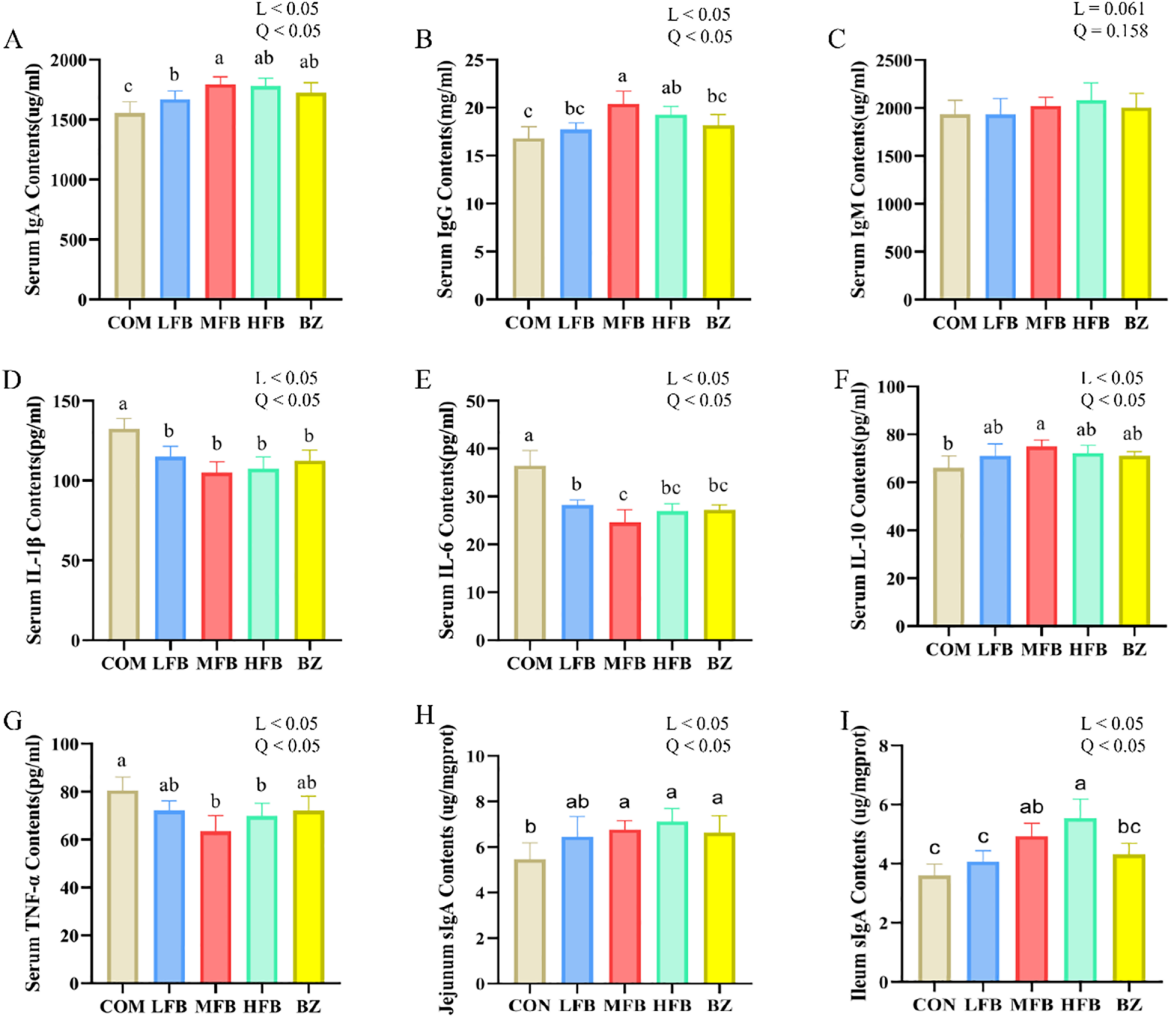
Effect of FB on the immune function of laying hens during the late laying period (n = 6). **(A)** Serum IgA contents; **(B)** Serum IgG contents; **(C)** Serum IgM contents; **(D)** Serum IL-1β contents; **(E)** Serum IL-6 contents; **(F)** Serum IL-10 contents; **(G)** Serum TNF-α contents; **(H)** Jejunum sIgA contents; **(I)** Ileum sIgA contents; CON, control group; LFB, group supplemented with 0.3% Bazhen San solid-state fermented product; MFB, group supplemented with 0.6% Bazhen San solid-state fermented product; HFB, group supplemented with 0.9% Bazhen San solid-state fermented product; BZ group, group supplemented with 0.6% unfermented Bazhen San. Different superscript letters (a–c) above the bar chart indicate significant differences (*P* < 0.05). L, P value of linear analysis; Q, P value of quadratic analysis.

The effects of FB on immune function in late-phase laying hens are shown in **Figure 1**. Compared with the CON group, serum IgA levels were significantly increased in all treatment groups (*P* < 0.05), whereas concentrations of IL-1β and IL-6 were significantly decreased (*P* < 0.05). Serum IgG levels were significantly increased in the MFB and HFB groups (*P* < 0.05). Notably, the MFB group exhibited a significantly higher IgG level than the BZ group (*P* < 0.05). There was no statistically significant difference in serum IgM levels among the groups (*P* > 0.05). As for cytokines, the MFB group showed a significantly higher serum IL-10 level than the CON group (*P* < 0.05). The TNF-α levels in both the MFB and HFB groups were significantly lower than those in the CON group (*P* < 0.05). Analysis of intestinal mucosal immunity revealed that jejunal sIgA content was significantly higher in the MFB, HFB, and BZ groups than in the CON group (*P* < 0.05); meanwhile, ileal sIgA levels were significantly increased in the MFB and HFB groups (*P* < 0.05). The results demonstrated that FB enhanced immune function (including humoral and intestinal mucosal immunity) and boosted anti-inflammatory capacity. Furthermore, the MFB group was more effective than the BZ group in elevating the levels of key immunomodulatory factors, IgG and IL-10.

### 3.5 The effect of FB on the intestinal antioxidant capacity of laying hens

**Figure 2:**
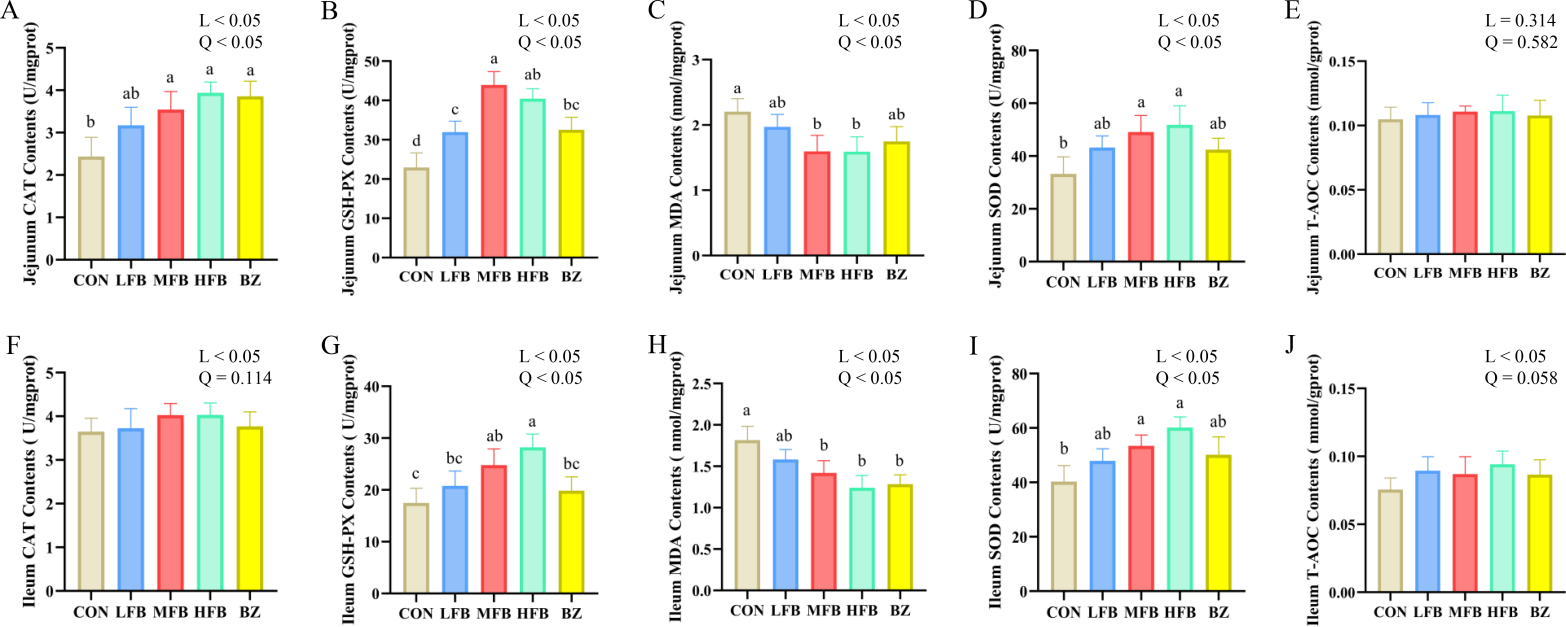
Effect of FB on intestinal antioxidant capacity of laying hens during the late laying period (n = 6). **(A)** Jejunum CAT contents; **(B)** Jejunum GSH-PX contents; **(C)** Jejunum MDA contents; **(D)** Jejunum SOD contents; **(E)** Jejunum T-AOC contents; **(F)** Ileum CAT contents; **(G)** Ileum GSH-PX contents; **(H)** Ileum MDA contents; **(I)** Ileum SOD contents; **(J)** Ileum T-AOC contents; CON, control group; LFB, group supplemented with 0.3% Bazhen San solid-state fermented product; MFB, group supplemented with 0.6% Bazhen San solid-state fermented product; HFB, group supplemented with 0.9% Bazhen San solid-state fermented product; BZ group, group supplemented with 0.6% unfermented Bazhen San. Different superscript letters (a–c) in the bar chart indicate significant differences (*P* < 0.05). L, P value of linear analysis; Q, P value of quadratic analysis.

The effects of FB on intestinal antioxidant capacity in late-phase laying hens are shown in **Figure 2**. Compared with the CON group, jejunal CAT activity was significantly increased in the MFB, HFB, and BZ groups (*P* < 0.05). Jejunal GSH-Px activity was significantly elevated in all treatment groups compared to the CON group (*P* < 0.05). Notably, the MFB group demonstrated significantly higher GSH-Px activity than the BZ group (*P* < 0.05). Meanwhile, the SOD activity in the jejunum of the MFB and HFB groups was significantly higher than that of the CON group (*P* < 0.05). Regarding oxidative damage, jejunal MDA concentration was significantly lower in the MFB and HFB groups than in the CON group (*P* < 0.05). In the ileal tissue, the GSH-Px activity in the MFB and HFB groups was significantly higher than that in the CON group (*P* < 0.05). Ileal MDA concentration was significantly lower in the MFB, HFB, and BZ groups than in the CON group (*P* < 0.05). In contrast, no significant differences were observed in the T-AOC of the jejunal and ileal mucosa, or in ileal CAT concentration, among the groups (*P* > 0.05). Comprehensive analysis reveals that the FB enhance key intestinal antioxidant enzyme activities and mitigate lipid peroxidation damage in laying hens, thus confirming their antioxidant potency. Medium and high doses demonstrated superior efficacy in strengthening the jejunal and ileal antioxidant defense system, suggesting a key role in mitigating oxidative stress and promoting intestinal health.

### 3.6 Morphological measurements of the jejunum and ileum

**Figure 3:**
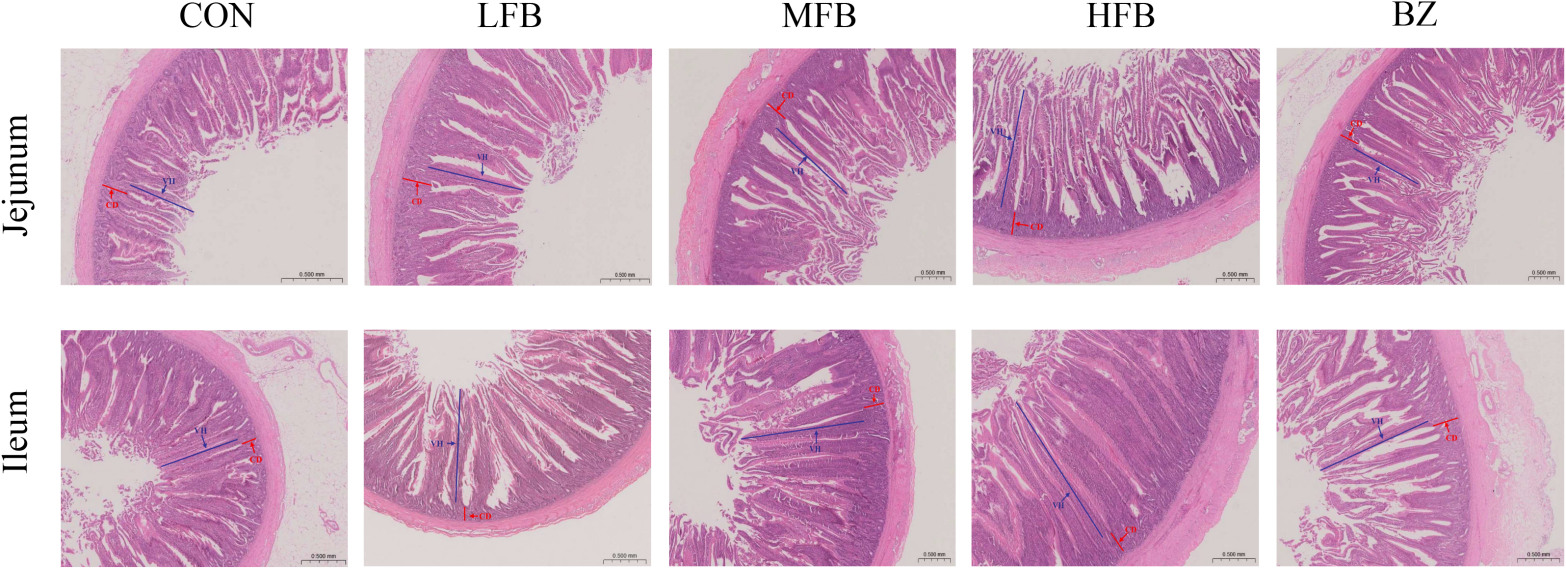
Effect of FB on intestinal morphology of laying hens during the late laying period (H&E staining, 50×) (n = 6). CON, control group; LFB, group supplemented with 0.3% Bazhen San solid-state fermented product; MFB, group supplemented with 0.6% Bazhen San solid-state fermented product; HFB, group supplemented with 0.9% Bazhen San solid-state fermented product; BZ group, group supplemented with 0.6% unfermented Bazhen San. VH: villus height; CD: crypt depth.

The results of the effects of FB on the intestinal morphology of laying hens during the late laying period are shown in **Figure 3** and **Table 4**. Compared with the CON group, jejunal villus height (VH) was significantly increased in all treatment groups (*P* < 0.05). The jejunal villus height/crypt depth (VH/CD) ratio was significantly increased in the MFB, HFB, and BZ groups compared to the CON group (*P* < 0.05). Regarding ileal morphology, the MFB, HFB, and BZ groups exhibited a significant increase in VH compared to the CON group (*P* < 0.05). Among them, the MFB group demonstrated a significantly greater VH than the BZ group (*P* < 0.05). In addition, the jejunal VH/CD ratio was significantly elevated in the MFB and HFB groups compared to that in the control group (*P* < 0.05). These results indicate that FB improves the intestinal morphology of laying hens. This improvement promotes nutrient absorption and thereby supports enhanced production performance.

**Table 4:** Effects of FB on intestinal morphology in laying hens during the late laying period.

Item	Group					SEM	P value		
	CON	LFB	MFB	HFB	BZ		A	L	Q
Jejunum									
VH(mm)	0.681^d^	1.045^c^	1.544^b^	1.747^a^	1.470^b^	0.081	<0.05	<0.05	<0.05
CD(mm)	0.178^b^	0.261^a^	0.239^a^	0.261^a^	0.205^ab^	0.009	<0.05	<0.05	<0.05
VH/CD	3.827^b^	4.068^b^	6.604^a^	6.835^a^	7.233^a^	0.343	<0.05	<0.05	<0.05
Ileum									
VH(mm)	1.131^d^	1.305^cd^	1.777^a^	1.572^b^	1.449^bc^	0.049	<0.05	<0.05	<0.05
CD(mm)	0.174^b^	0.165^b^	0.232^ab^	0.182^bc^	0.266^a^	0.009	<0.05	0.240	0.245
VH/CD	6.535^bc^	8.006^ab^	7.871^ab^	8.675^a^	5.485^c^	0.284	<0.05	<0.05	<0.05

Data were expressed as means ± SEM (n = 6).

SEM: Pooled standard error of the mean.

VH, villus height; CD, crypt depth; VH/CD, villus height/crypt depth.

^a–d^Means within each row with different superscripts are statistically significantly different (*P* < 0.05).

A, P value of one-way ANOVA; L, P value of linear analysis; Q, P value of quadratic analysis.

### 3.7 The effect of FB on intestinal mucosal barrier function in laying hens

**Figure 4:**
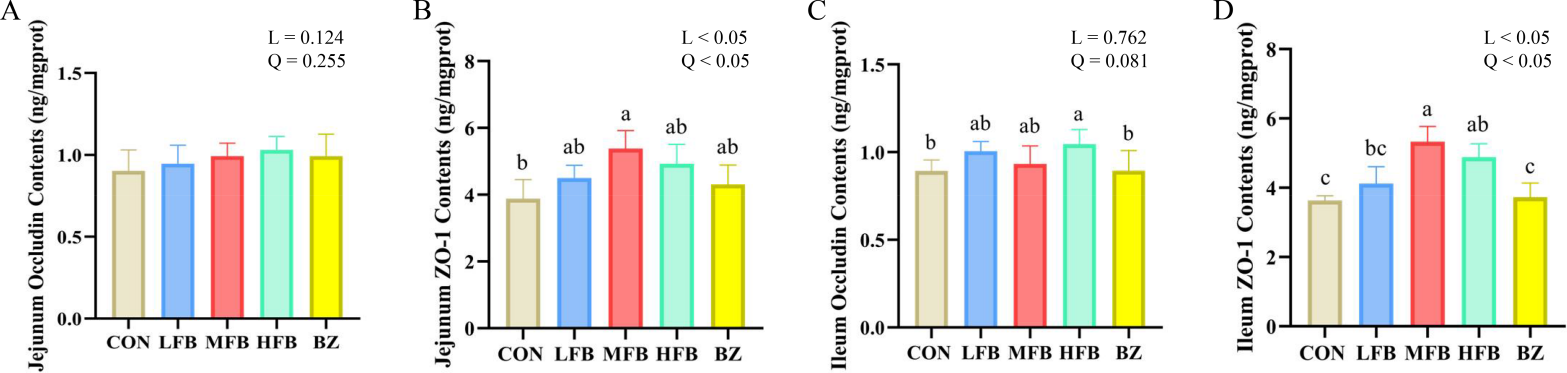
Effect of FB on intestinal tight junction protein (Occludin) and zonula occludens-1 (ZO-1) concentrations in laying hens during the late laying period (n = 6). **(A)** Jejunum Occludin contents; **(B)** Jejunum ZO-1 contents; **(C)** Ileum Occludin contents; **(D)** Ileum ZO-1 contents; CON, control group; LFB, group supplemented with 0.3% Bazhen San solid-state fermented product; MFB, group supplemented with 0.6% Bazhen San solid-state fermented product; HFB, group supplemented with 0.9% Bazhen San solid-state fermented product; BZ group, group supplemented with 0.6% unfermented Bazhen San. Different superscript letters (a–c) in the bar chart indicate significant differences (*P* < 0.05). L, P value of linear analysis; Q, P value of quadratic analysis.

The effects of FB on the intestinal mucosal barrier function in late-phase laying hens are shown in **Figure 4**. Compared with the CON group, the concentration of ZO-1 in the jejunal mucosa was significantly increased in the MFB group (*P* < 0.05). There was no statistically significant difference in the concentration of Occludin in the jejunum among the treatment groups (*P* > 0.05). The Occludin concentration in the ileal mucosa was significantly higher in the HFB group than in the CON group (*P* < 0.05). The ZO-1 concentration in the ileal mucosa of the MFB group was significantly higher than that in the CON and BZ groups (*P* < 0.05). These results indicate that FB improves intestinal integrity.

### 3.8 Effects of FB on the gut microbiota of laying hens

To evaluate the effects of dietary supplementation with FB on gut microbiota, 16S rRNA gene sequencing was performed on cecal content samples from late-phase laying hens. A total of 30 cecal content samples were subjected to sequencing. After filtering 3,425,898 raw tags, 3,395,051 clean tags were obtained.

**Figure 5:**
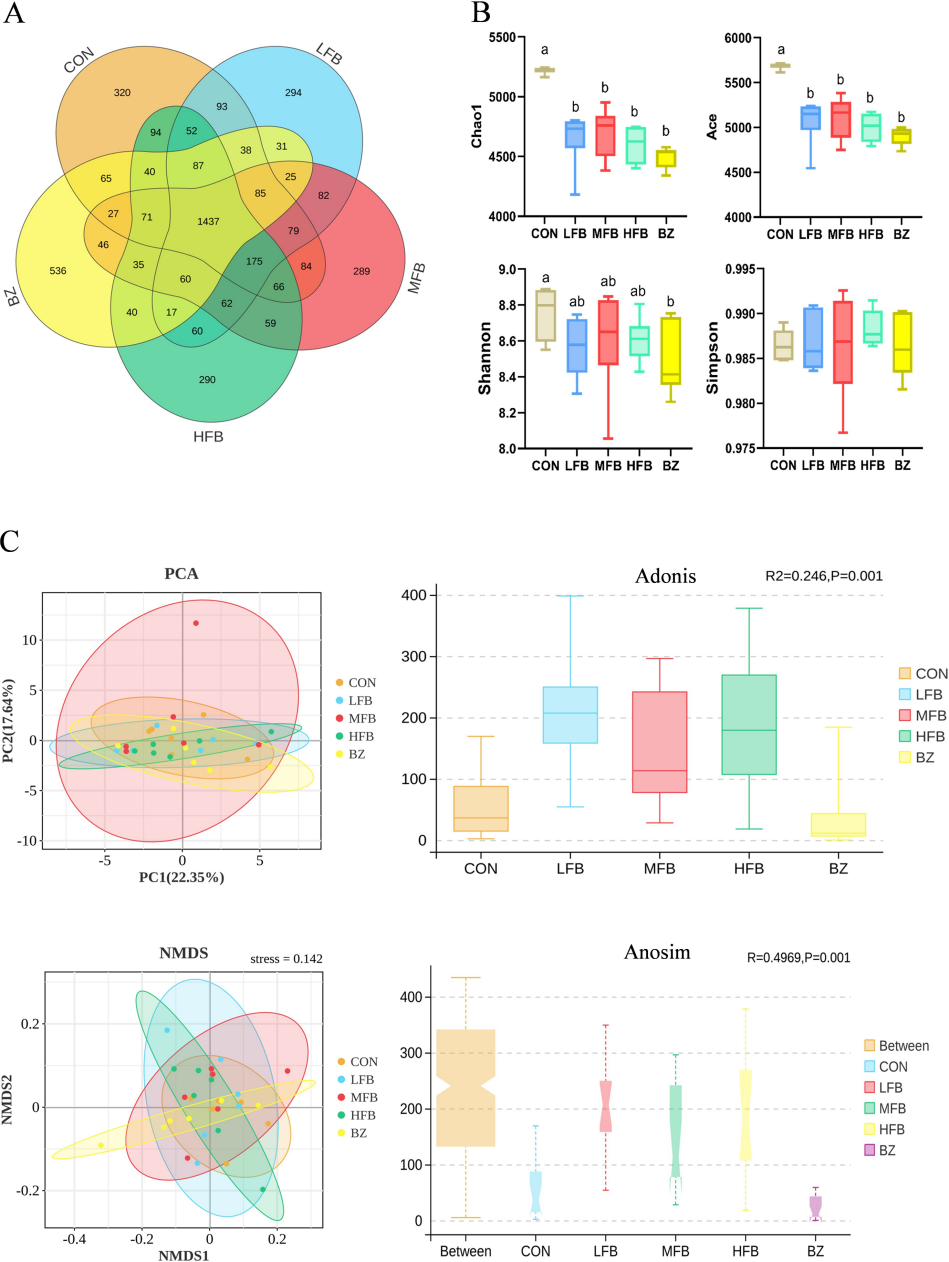
Effect of FB on the diversity of cecal microbiota in laying hens during the late stage of egg production (n = 6). CON, control group; LFB, 0.3% solid-state fermented Bazhen San supplemented group; MFB, 0.6% solid-state fermented Bazhen San supplemented group; HFB, 0.9% solid-state fermented Bazhen San supplemented group; BZ, 0.6% non-fermented Bazhen San supplemented group. **(A)** OTU-level Venn diagram. **(B)** Alpha diversity analysis, including Chao1, ACE, Shannon, and Simpson indices. **(C)** Beta diversity analysis, PCA and NMDS analyses.

The Venn diagram based on OTU statistics is shown in **Figure 5A**. After the 8-week dietary intervention, the cecal contents of CON, LFB, MFB, HFB, and BZ groups contained 2,813, 2,675, 2,682, 2,645, and 2,640 OTUs, respectively. Among these, 1,437 OTUs were shared across all five groups, while the numbers of unique OTUs in the CON, LFB, MFB, HFB, and BZ groups were 320, 294, 289, 290, and 536, respectively. The results demonstrated that supplementation with FB and BZ during the late laying period of laying hens influenced the microbial composition in the cecal microbiota of the birds.

The alpha diversity analysis results are shown in **Figure 5B**. The Chao1 and Ace indices represent the community richness of the samples, while the Shannon index reflects the community diversity and evenness, and the Simpson index indicates the dominance of abundant species in the community. Compared with CON group, the Chao1 and Ace indices of the cecal microbiota in the LFB, MFB, HFB, and BZ groups were significantly reduced (*P* < 0.05). The Shannon index in the BZ group was significantly lower than that in the CON group (*P* < 0.05), whereas no significant differences were observed in the FB-fed groups (*P* > 0.05). Similarly, there were no significant differences in the Simpson index among the treatment groups compared to the CON group (*P* > 0.05).

The beta diversity analysis results are shown in **Figure 5C**. Principal Component Analysis (PCA) revealed distinct separation states between the control group (CON) and experimental groups. Compared to the CON group, the cecal microbiota of the other groups exhibited a separation trend, with PC1 accounting for 22.35% and PC2 for 17.64% of the variance. The unweighted UniFrac distance algorithm was employed to evaluate the statistical significance of structural changes in the intestinal microbial communities of laying hens using Adonis tests. The results demonstrated significant alterations (*P* < 0.05) in microbial community structure for the comparisons CON *vs* MFB, CON *vs* HFB, CON *vs* BZ, LFB *vs*-BZ, MFB-*vs*-BZ, and HFB-*vs*-BZ. Non-metric Multidimensional Scaling (NMDS) and Adonis tests further confirmed that microbial composition at the OTU level was significantly separated (*P* < 0.05) in these pairwise comparisons, with intergroup differences substantially greater than intragroup variations.

**Figure 6:**
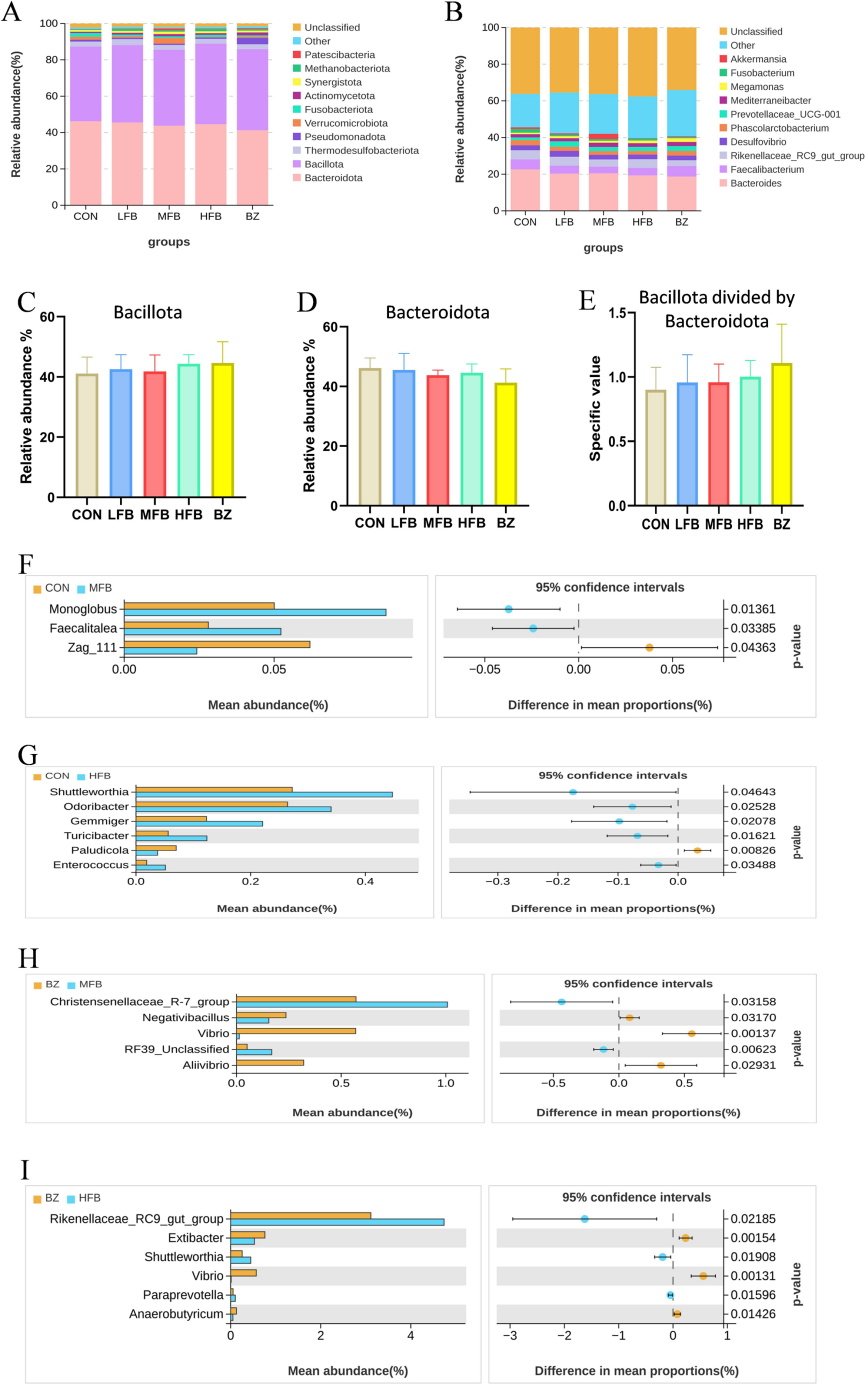
The effect of FB on the composition of cecal microbiota in laying hens during the late stage of egg production (n = 6). CON, control group; LFB, 0.3% solid-state fermented Bazhen San supplemented group; MFB, 0.6% solid-state fermented Bazhen San supplemented group; HFB, 0.9% solid-state fermented Bazhen San supplemented group; BZ, 0.6% non-fermented Bazhen San supplemented group. **(A)** Phylum-level taxonomic composition analysis. **(B)** Genus-level taxonomic composition analysis. **(C)** Relative abundance of Bacteroidetes. **(D)** Relative abundance of Firmicutes. **(E)** Firmicutes/Bacteroidetes (F/B) ratio. **(F–I)** Results of Welch´st-test.

The effects of FB supplementation on cecal microbiota composition in laying hens during the late laying period are shown in **Figure 6**. As illustrated in **Figure 6A**, at the phylum level, the cecal microbiota of all five treatment groups was predominantly composed of *Bacteroidota* and *Firmicutes*, collectively accounting for over 80% of the total relative abundance. Several other phyla, including *Thermodesulfobacteriota*, *Pseudomonadota*, *Verrucomicrobiota*, *Fusobacteriota*, *Actinomycetota*, *Synergistota*, *Methanobacteriota*, and *Patescibacteria*, were present at lower abundances. **Figures 6C-E**- demonstrate that compared to the CON group, no significant differences (*P* > 0.05) were observed in the relative abundances of *Bacteroidota*, *Firmicutes*, or the *Firmicutes*-to-*Bacteroidota* ratio across the treatment groups.

As shown in **Figure 6B**, at the genus level, the predominant taxa included Bacteroides, *Faecalibacterium*, *Rikenellaceae_RC9_gut_group*, *Desulfovibrio*, *Phascolarctobacterium*, *Prevotellaceae_UCG-001*, *Mediterraneibacter*, *Megamonas*, *Fusobacterium*, and *Akkermansia*. Welch’s t-test results (**Figures 6F–I**) revealed significant differences in bacterial abundances at the genus level among groups. Compared to the CON group, the MFB group exhibited increased abundances of *Monoglobus* and *Faecalitalea*, while the HFB group showed elevated abundances of *Shuttleworthia*, *Odoribacter*, *Gemmiger*, *Turicibacter*, and *Enterococcus*. Compared with the BZ group, the MFB group demonstrated higher abundances of Christensenellaceae_R-7_group and RF39_Unclassified. In contrast, the BZ group displayed a higher abundance of *Negativibacillus*, *Vibrio*, and *Aliivibrio* than the MFB group. The HFB group was enriched in Rikenellaceae_RC9_gut_group, *Shuttleworthia*, and *Paraprevotella*, whereas the BZ group exhibited higher abundances of *Extibacter*, *Vibrio*, and *Anaerobutyricum* compared to the HFB group.

### 3.9 Spearman correlation analysis

**Figure 7:**
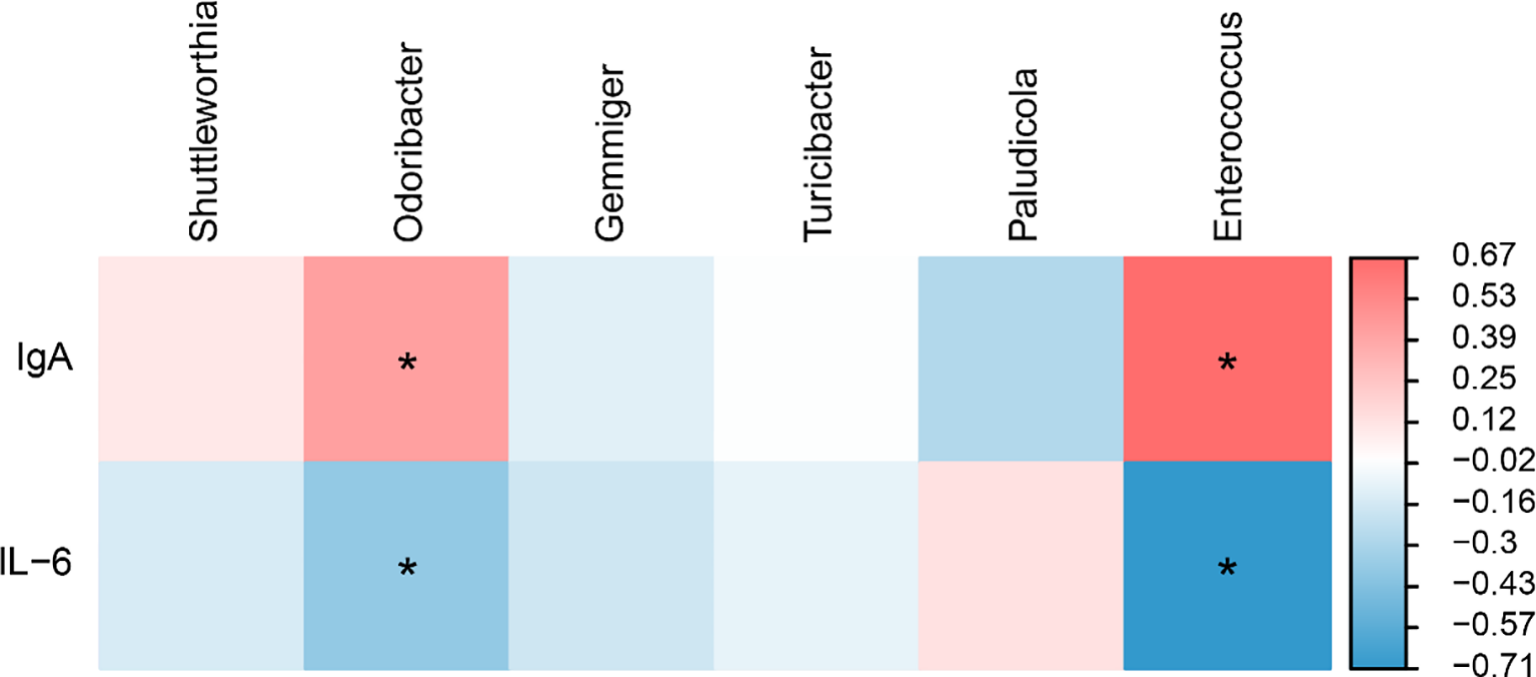
Heatmap of the adjusted analysis between cecal microbiota differences and serum immunity (n = 6). Red indicates positive correlations, while blue denotes negative correlations. * represents correlations with significance (*P* < 0.05).

To explore associations between the gut microbiota and immunity, a Spearman correlation analysis of differential bacterial genera and immune parameters was performed, and the results are presented in **Figure 7**. The Spearman correlation matrix revealed that the relative abundance of *Odoribacter* and *Enterococcus* was positively correlated with serum IgA levels and negatively correlated with serum IL-6 concentrations (*P* < 0.05). These correlation results suggest that *Odoribacter* and *Enterococcus* may play a beneficial role in modulating host immunity.

## 4 Discussion

The decline in production performance during the late laying phase is a complex issue linked to a comprehensive decline in physiological functions, particularly immune dysregulation and impaired intestinal health (6, 19). This study demonstrates that FB improves the production performance of laying hens by reshaping intestinal microbiota, repairing the intestinal barrier, and modulating immune function, with effects significantly superior to those of the BZ.

This study found that both FB and BZ increased the egg production rate and reduced the feed-to-egg ratio, indicating that the base formula itself improves the health and production performance of laying hens during the late laying phase. However, the MFB group demonstrated significant superiority over the BZ group by more effectively reducing the feed-to-egg ratio, improving yolk color, and enhancing humoral immunity (IgG). These findings provide key evidence for the value-added effect of the fermentation process. This advantage is likely attributable to three mechanisms. First, fermentation degrades plant cell walls, thereby increasing the content of active ingredients (e.g., polysaccharides and flavonoids) and breaking down large molecules into smaller ones to enhance bioavailability (20–22). Second, the process produces metabolites (such as lactic acid) that participate in immune regulation and exhibit synergistic effects with the herbal compounds (21, 23). Finally, fermentation reduces potential antinutritional factors in BZ, further improving its bioavailability (24).

The intestine serves as the primary site for nutrient absorption, and its structural integrity is fundamental to optimal production efficiency (25). The MFB and HFB groups demonstrated significantly increased VH and VH/CD ratio in the jejunum and ileum. This indicates that FB effectively expanded the intestinal surface area for nutrient absorption and promoted the healthy maturation of the intestinal mucosa (26–28). More importantly, FB significantly upregulated the expression of key tight junction proteins ZO-1 and Occludin in the jejunum and ileum. As core structural proteins that maintain tight junction integrity, the enhanced expression of ZO-1 and Occludin strengthens the intestinal physical barrier function (29, 30). An intact intestinal barrier effectively prevents the translocation of endotoxins and pathogens into systemic circulation, a key mechanism for controlling systemic inflammation (31). This discovery provides a direct mechanistic link explaining the observed systemic reduction in pro-inflammatory cytokine levels, as detailed in the subsequent analysis of immune indicators.

Concurrent with the enhancement of the physical barrier, sIgA levels in the jejunum and ileum were significantly increased in the MFB and HFB groups, indicating that FB also enhanced the local intestinal immune barrier. The elevated sIgA levels signify a strengthened mucosal immune defense, which enhances the capacity to neutralize intestinal pathogens and toxins (32–34). Furthermore, sIgA also strengthens the intestinal barrier by facilitating the adhesion and colonization of beneficial bacteria (34). In addition, FB treatment significantly increased the activities of intestinal antioxidant enzymes (GSH-Px, SOD) and reduced the content of the lipid peroxidation product, malondialdehyde (MDA). This effect can be attributed to the antioxidant activity of inherent constituents in Bazhen San, such as polysaccharides and flavonoids (35, 36). Moreover, the fermentation process may enhance the efficacy of these constituents or generate novel antioxidants, thereby more effectively alleviating oxidative stress in the late laying phase (37, 38).

The enhancement of local intestinal health by the FB was correspondingly reflected in improved systemic immune status. Specifically, FB treatment significantly elevated serum levels of IgA and IgG, while concurrently reducing the concentrations of pro-inflammatory cytokines IL-1β and IL-6. As key effectors, IgA protects mucosal surfaces from pathogen invasion (39), and IgG is a major circulating antibody involved in systemic immune responses (40). Conversely, IL-1β is a key mediator of chronic inflammation (41), and IL-6 is a pleiotropic cytokine produced in response to inflammatory stimuli (42). These immunological improvements—elevated antibody levels and reduced pro-inflammatory cytokines—are crucial for alleviating immune stress in late-phase laying hens, thereby enhancing overall flock health and production performance (43, 44). Furthermore, the MFB group demonstrated a significantly greater increase in serum IgG than the BZ group, an enhancement attributable to the synergistic effects of the fermentation process (37).

The microbiome analysis of the cecum provides mechanistic insights underlying the enhanced therapeutic efficacy of FB. The active ingredients in Bazhen San are not only direct immune-active substances (45, 46) but also serve as important carbon sources and prebiotics for the gut microbiota (47, 48). The solid-state fermentation process acts as a pre-digestion of the Bazhen San formulation. Fermentation microorganisms degrade these complex plant components into oligosaccharides and small molecules that are more easily utilized by the host’s gut microbiota (49, 50). This biotransformation creates a microenvironment that promotes the proliferation of beneficial bacterial communities, thereby explaining the observed structural changes in the gut microbiota. Compared with the CON group, the abundance of *Monoglobus* and *Faecalitalea* was increased in the MFB group. These genera are known to possess immunomodulatory and anti-inflammatory functions and to synthesize short-chain fatty acids (SCFAs) (51, 52). The HFB group exhibited an increased relative abundance of *Shuttleworthia*, *Odoribacter*, *Gemmiger*, *Turicibacter*, and *Enterococcus*. Spearman correlation analysis revealed that the relative abundances of *Odoribacter* and *Enterococcus* were positively correlated with serum IgA levels and negatively correlated with IL-6 concentration. Previous studies have shown that *Odoribacter* plays a crucial role in regulating immunity and improving intestinal barrier function (53, 54), while *Enterococcus* exhibits anti-inflammatory and antipathogenic effects (55). These findings elucidate the mechanism of FB’s immunoregulatory effects from a gut microbiota perspective. Therefore, dietary supplementation with FB in the late-stage diet of laying hens can modulate the intestinal abundance of *Odoribacter* and *Enterococcus*, thereby enhancing immunity and anti-inflammatory capacity.

## 5 Conclusions and prospects

In summary, dietary supplementation with FB enhanced the laying performance, egg quality, immune function, intestinal antioxidant capacity, and morphological structure of the intestine in late-phase laying hens. Furthermore, it increased the relative abundance of beneficial bacteria in the cecal microbiota. The supplementation of 0.6% and 0.9% FB was most effective in improving immunity and intestinal health. This research indicates that FB has the potential to serve as a functional feed additive for improving the health and productivity of late-phase laying hens.

A limitation of this study is that the active components of FB, including key metabolites, and their mechanisms *in vivo* of action have not been fully characterized. Future research will focus on the following three directions: First, to systematically identify the active ingredients in the FB fermentation product and their distribution in the bloodstream; Second, to validate the changes in key metabolites using metabolomics; Third, to elucidate the specific pathways regulating immunity through molecular biology approaches. These efforts will provide deeper mechanistic evidence to support the development and application of FB.

## Data Availability

The original contributions presented in the study are included in the article/supplementary material. Further inquiries can be directed to the corresponding authors.
